# Crimean-Congo hemorrhagic fever, a real health problem in Iraq?

**DOI:** 10.1016/j.ijregi.2025.100588

**Published:** 2025-01-31

**Authors:** Masood Abdulkareem Abdulrahman

**Affiliations:** Department of Public Health, College of Health and Medical Technology-Shekhan, Duhok Polytechnic University, Duhok, Iraq

**Keywords:** Crimean-Congo hemorrhagic fever (CCHF), Outbreak, 2023, Iraq

## Abstract

**Objectives:**

Crimean-Congo hemorrhagic fever (CCHF) is the most widespread tick-borne viral disease worldwide. CCHF was not recognized in Iraq before 1979, after which many outbreaks occurred, and the disease became endemic with the re-emergence of outbreaks. This study aimed to analyze the epidemiology of the largest outbreak in Iraqi history in 2023.

**Methods:**

This retrospective study included human CCHF cases from 2023.

**Results:**

2186 suspected cases were investigated. There were 587 confirmed cases, and 83 deaths, and an overall case fatality rate (14%). Among the confirmed cases, only 539 cases had complete data, and the analysis was performed on these cases. The majority 70.9% of patients lived in the southern provinces. Approximately 58% of the patients were male and up to half of the patients resided in rural areas. Approximately 45% of cases were in the 25-44 years age group. The occupations of the patients were as follows: 30% were housewives, 22% were butchers, 18% were animal owners, 30% had other occupations, and up to 60% had a history of exposure to fresh raw meat.

**Conclusions:**

The 2023 outbreak was the largest in Iraq in decades. The absence of preventive and control activities during the COVID-19 pandemic played an important role in the rise of cases and the presence of unlicensed and freelance slaughterers, especially during religious events played an important role in this epidemic.

## Highlights

• Crimean-Congo hemorrhagic fever (CCHF) infection is the most widespread, tick-borne viral disease.
• The first CCHF outbreak in Iraq was recognized in Iraq September 1979.
• The CCHF outbreak in 2023 was the biggest in Iraq in decades.
• The absence of preventive and control activities during the COVID-19 pandemic has played an important role in the increase in cases.

## Introduction

Human Crimean-Congo hemorrhagic fever (CCHF) infection is caused by a virus Class- Bunaviricetes; Order- Hareavirales; Family - Nairovirus, Genus- Orthonairvirus. It mainly occurs after the bite of an infected tick of Hyalomma spp. or exposure to blood or tissues from infected animals; human-to-human transmission, especially among healthcare workers, can occur [1]. Case fatality rates (CFR) during outbreaks may reach 40%. The CCHF is endemic to Africa, the Balkans, the Middle East (including Iraq), northwestern China, central Asia, southern Europe, and the Indian subcontinent south of the 50th parallel north – the main tick vector's geographical range [2]. World Health Organization (WHO) estimates that more than three billion people are at risk of developing CCHF, and the annual incidence of CCHF is 10,000-15,000 cases [3], which may be due to multiple causes, such as climate change and an increase in the vector number, the advancement of viral detection methods, and rising exposure of humans and animals [4]. Only humans become ill after acquiring the CCHF virus (CCHFV). In an endemic transmission cycle, a range of animals might serve as CCHFV reservoirs without displaying any symptoms [5]. Currently, CCHF is known to be endemic or presumably endemic to more than 50 countries in Europe, Africa, and Asia. It has been linked to severe hemorrhagic syndrome in humans and sporadic infections in travelers visiting these regions and may infect both domestic and wild animals in the absence of any particular clinical manifestations [6]. In nature, CCHFV often follows an enzootic tick-vertebrate-tick cycle.

When Soviet troops reoccupied the German-occupied portions of the Crimean Peninsula in the mid-1944s, the first known epidemic of CCHFV occurred. Acute febrile sickness with a high rate of shock and hemorrhage affected approximately 200 Soviet soldiers [1]. In 1956, the disease was isolated from Belgian Congo (now known as the Democratic Republic of the Congo, formerly Zaire). In 1969, researchers discovered that viruses isolated in 1944 and 1956 were identical [7]. By combining the names of the two locations, sickness, and virus are now known as Crimean-Congo hemorrhagic fever [7].

CCHF was unrecognized in Iraq before September 1979 when a pregnant woman from Ramadi city was admitted to Al Yarmouk Teaching Hospital in the Baghdad capital of Iraq, on 7th September, 1979 with hemorrhagic manifestations, and she died 2 days later [8]. After the first CCHF outbreak in Iraq, many outbreaks occurred, and the disease became endemic with the re-emergence of outbreaks [9]. Few studies have investigated the distribution and species of Hyalomma ticks in Iraq. A review study done by Abed and Alhaboubi on Hyalomma infestation on animals in Iraq, they found about 13 species of the ixodid tick of two genera Rhipicephalus, Hyalomma are recorded in Iraq that transmits a wide range of etiological agent. As a multi-host species, ticks are considered a major challenge in enhancing livestock health and productivity, especially in developing countries. Hyalomma spp. assign their veterinary significance to the high ability to transmit the hemoprotozoan parasite of Babesia and thieleria species [10]. Another study done by Khwarahm predicted and mapped the geographical distribution of the key vector ticks responsible for the transmission of CCHF disease in Iraq. He found that in Iraq, Only a small portion of its geographical area— approximately 51% of the total area, or 225,665 km^2^ out of 441,724 km^2^—provides an adequate habitat for the species. Most of these potential areas (approximately 41.57% or 183, 631 km^2^) were considered to be of low suitability. In total, 8.61% (38,039 km^2^) of the region is moderately suitable, whereas only 0.9% (3994 km^2^) was highly suitable. The southern provinces of Dhi-Qar, Mysan, and Basra are among the areas most at risk of CCHF spread. Furthermore, as the modeling showed, certain districts of the capital city of Baghdad were identified as hazardous locations for the growth of vector ticks. Moderately risky regions encompass certain sites across Diyala, Wasit, and Almuthanna in the southern part of Iraq, and small areas in Duhok, Erbil, Kirkuk, and Ninawa in the north. Notably, significant portions of the country, particularly the north, northeast, and western areas of Anbar, were found to have unsuitable habitats for Hyalomma vector ticks in Iraq. Overall, the southern provinces, in comparison to the northeastern provinces, seem more suitable to the hard ticks to complete their life cycles [11]. Accordingly, this study was conducted to analyze the epidemiology of the last largest outbreak in Iraqi history in 2023.

## Methodology

This is a retrospective study done for human CCHF cases during 2023 in Iraq. These cases were reported to the Iraqi Office of the WHO by authorities of the Federal Ministry of Health, Iraq. Iraq has 18 provinces, three of them in the Kurdistan Region (Erbil, Duhok, and Sulaymaniyah) in the north of the country, the rest of the provinces are in the middle and south, total population of 2024 of (46,013,046), the country land in square kilometer (434,128) [12]. Based on WHO resources, the clinical manifestations of CCHF range from asymptomatic (<88%) infection or mild, nonspecific febrile illness to severe hemorrhagic disease with multiorgan failure leading to death. CCHF case definitions vary across endemic regions; the case definition proposed by Ergonul et al. includes suspect, probable, and confirmed cases [13,14]. All CCHFV-suspected patients who showed these clinical manifestations were hospitalized in separate CCHFV wards and Intensive Care Units. Blood samples were collected from all patients reported during the outbreak. The samples were sent immediately to the Central Public Health Laboratory (Baghdad) and handled under maximum biological containment conditions because the CCHF patient samples presented an extreme biohazard risk. All confirmed cases of CCHF were diagnosed using reverse transcription polymerase chain reaction (RT-PCR). To ensure the safety of specimen handling, serum samples were inactivated before nucleic acid extraction and amplification. Following the guidelines provided by the manufacturer (Geneaid, South Korea), viral RNA was extracted from serum samples. The WHO-validated kit (CCHFV RNA, validated for Eurasian clades IV-VII) was used to determine the RT-PCR oligonucleotide sequences and primers for the detection of CCHFV genes. For current infections, the RT-PCR test provides the greatest detection sensitivity at the earliest feasible moment [15].

### Data collection process

Three types of data sources were used. The first included case investigation forms for all suspected cases that were received at the zoonotic disease section of the Directorate of Preventive Affairs in each province. The second set included the case sheets of all confirmed cases admitted to hospitals in all Iraqi provinces. These case sheets were retrieved from hospitals after approval was granted to all concerned bodies at the central and provincial levels. The third data source included laboratory results received from the Central Public Health Laboratory tests including liver enzyme tests, white blood cell count, platelet count, blood film, blood urea, and all blood samples from suspected cases were tested for CCHFV by using the real-time RT-PCR test.

The variables included in the case investigation forms were selected based on the case definition adopted by the Iraq Center for Disease Control (CDC). The variables collected included the reporting directorate for health, time of reporting, primary diagnosis, demographic characteristics of patients (patients’ age, sex, and residence), main signs and symptoms, date of symptom onset, investigation results, history of exposure, and risk factors, including (residency in rural areas, tick exposure, contact with animals, direct contact with uncooked meat, contact with similar cases, slaughtering of the animals at home in the last 2 weeks, and occupation). Data analysis was performed by the Directorate of the Centers for CDC and Prevention at the Federal Ministry of Health, Baghdad.

## Results

**Figure 1:**
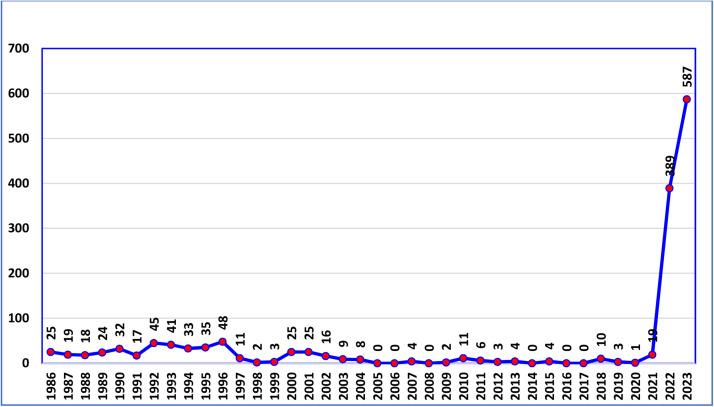
Trend of Crimean-Congo hemorrhagic fever cases in Iraq since 1986.

Figure 1 shows the trend in confirmed cases of CCHF from 1986 to 2023. The disease trend from 1986 to 2021 oscillated especially from zero cases (2005, 2006, 2014, 2016) to a maximum of 48 cases in 1996. However, a sharp increase was noticed in the last 2 years to 389 cases in 2022, and a peak was observed in 2023 (587) confirmed cases. Figure 2a shows the distribution of cases according to provinces in 2022, which were disturbed in all provinces except for Sulaymaniyah Province, with the largest number of cases reported in Thiqar Province (162). Figure 2b shows the distribution of cases according to the provinces in 2023, the cases were distributed to all provinces 70.9% (416) of the confirmed cases were in the southern provinces, while only 8.2% (48) cases were in the five northern provinces (Kirkuk, Ninawa, Erbil, Duhok, and Sulaymaniyah).

**Figure 2a:**
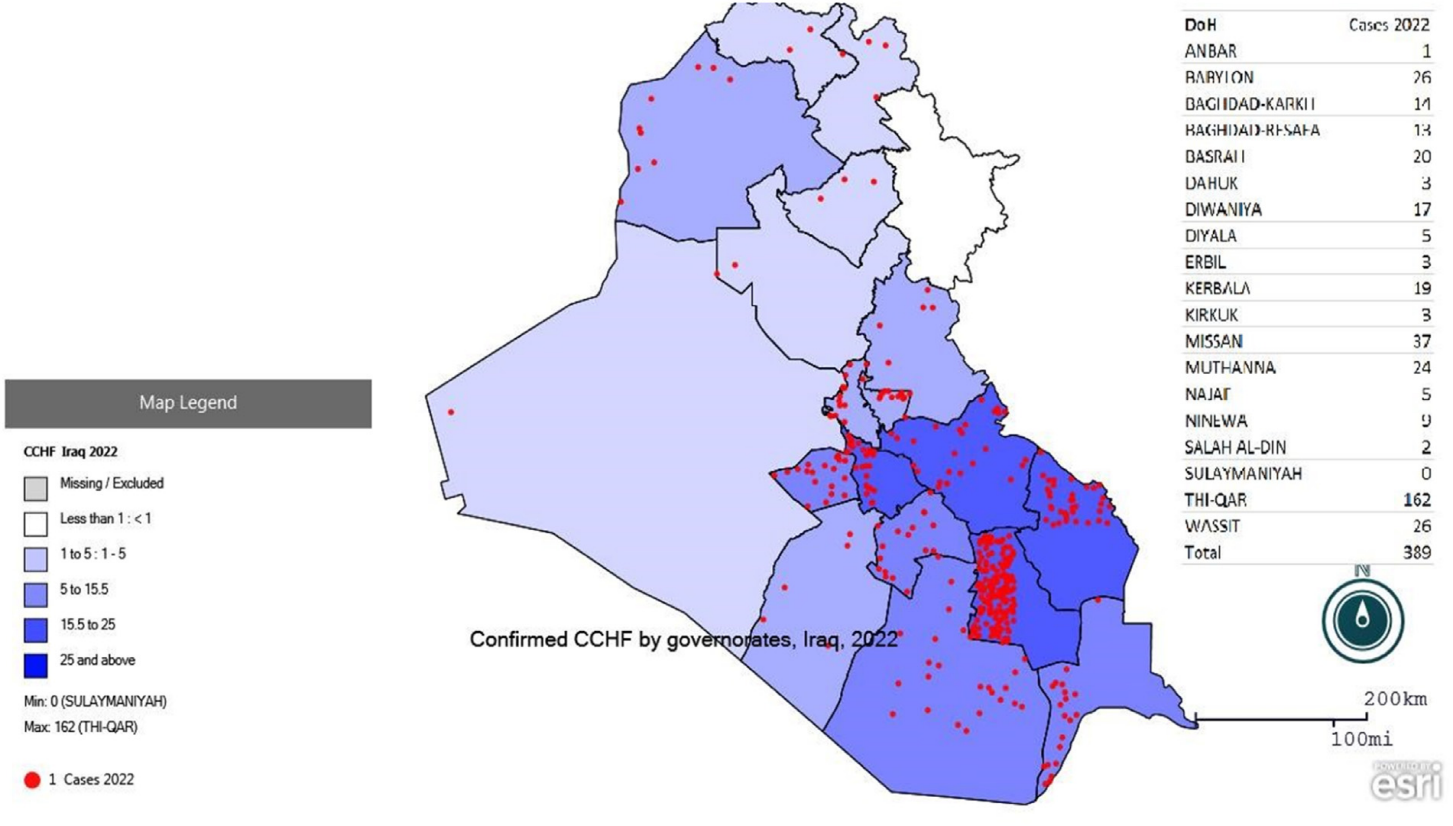
Distribution of CCHF cases among Iraqi provinces during 2022.

**Figure 2b:**
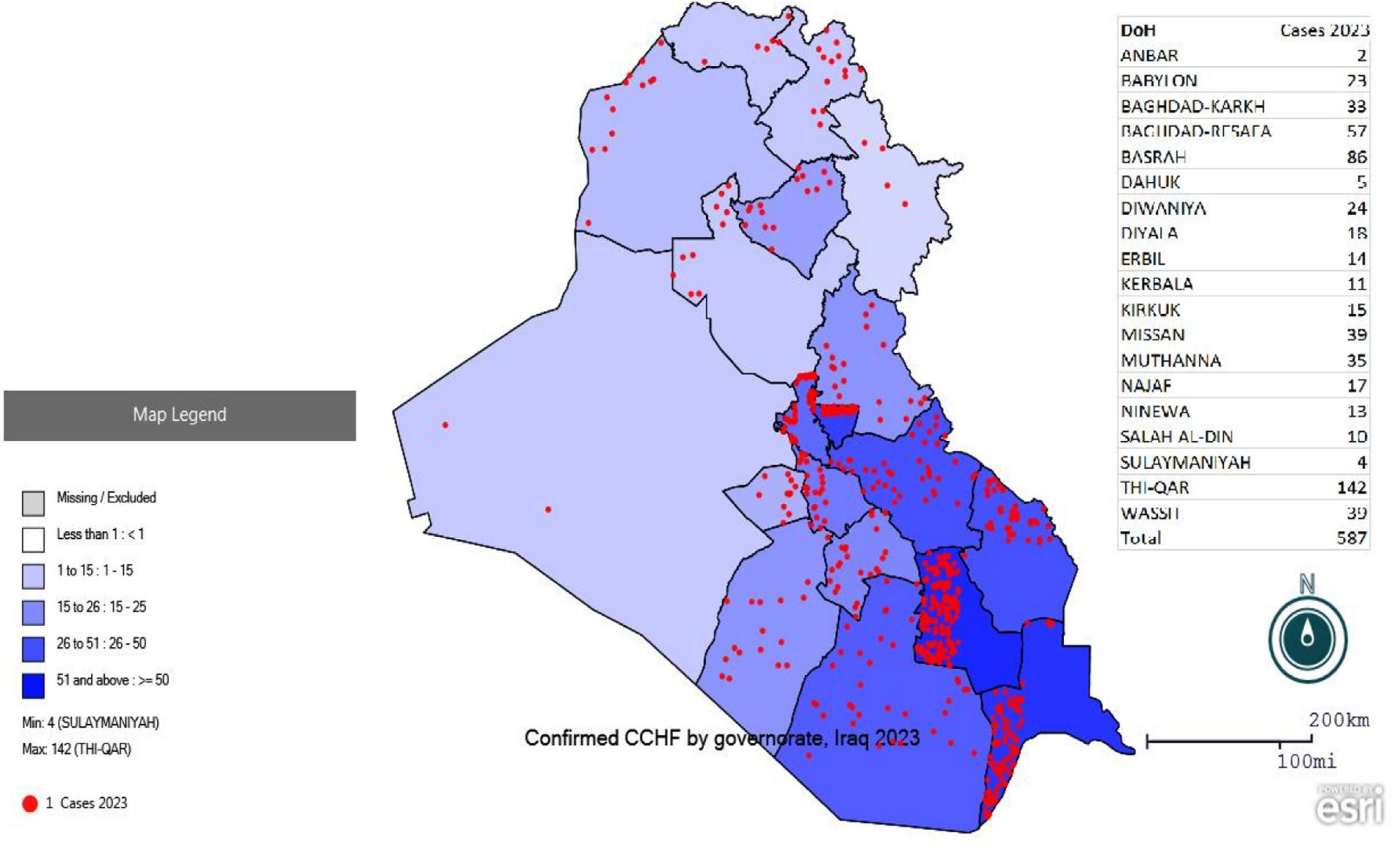
Distribution of CCHF cases among Iraqi provinces during 2023. CCHF, Crimean-Congo hemorrhagic fever.

At the end of week 52 - 2023, The federal MoH reported and investigated 2186 suspected cases, and 144 deaths occurred among suspected patients with CFR (7%). There were 587 confirmed cases, and 83 deaths, and an overall CFR (14%). Among 587 confirmed cases, only 539 cases had complete data; therefore, the analysis was performed for 539 cases. Approximately 58% (n = 313) of the patients were male and 42% (n = 226) were female. Up to half of the patients were residents of rural areas, 40% were residents of urban areas, and the rest were residents of semi-urban areas. Regarding the age groups, about 45% of cases are in the 25-44 years age group, which is the most economically active age group, 24% of the 45-64 years age group, in 22% of the 15-24 years age group, 6% were old age ≥65 years, the least 3% of 5-14 years age group. The occupations of the patients were as follows: 37% were housewives, 12% were butchers, 10% were students, 9% were animal owners, 8% were public employers, 1% were healthcare workers, and 23% had other occupations. The confirmed cases had a history of exposure to one or two risk factors as follows: 328 cases had a history of exposure to fresh raw meat, 287 cases had a slaughtering history, 282 had a history of the presence of animals at home, 43 cases had a history of tick bite, and 15 of cases had a history of contact with suspected or confirmed cases.

## Discussion

CCHF is the most common tick-borne viral hemorrhagic fever, both in Iraq and worldwide. The number of confirmed cases of CCHF fluctuated from 1986 to 2021, ranging from zero in 2005, 2006, 2014, and 2016 to a peak of 48 in 1996. However, a sharp increase was noticed in the last 2 years to 389 cases in 2022, and a peak was observed in 2023 (587) confirmed cases. The reported cases increased from 1989 (24 cases) to 1996 (48 cases), which may be due to sequelae of the second Gulf War (1990-1991) and followed by international sanctions on Iraq; 4 days after the Iraqi invasion of Kuwait, the United Nations Security Council (UNSC) placed a comprehensive embargo on Iraq, which led to the deterioration of most of the health services in Iraq [16]. Following United Nations Security Council resolution 986 (1995), Many UN agencies established many humanitarian actions, such as financing the export of medicine, health supplies, foodstuffs, and materials and supplies for essential civilian needs. The Food Agriculture Organization (FAO) implemented a 3-year agricultural program in coordination with the Ministry of Agriculture, including pest control programs. In 1996, tick control campaigns in Iraq resulted in a decrease in the number of CCHF cases annually. Between 1997 and 1999, the annual number of cases ranged from 11 to three, while in 2010, it increased to 11 owing to a lack of compliance with tick control activities. This reflects the actual need for regular laboratory investigations for the presence of infection in livestock and frequent checking for the extent of livestock tick infestation with the application of suitable immediate preventive measures such as cattle and barn spraying with sheep and goat dipping [17]. In 2016, a serostudy was conducted in Iraq, Iran, and Turkey to investigate the disease animal host. The results showed that in Iraq, 20-30% of the animals tested positive, with goats having the highest percentage (50%) of positive animals [5]. The rise in CCHF instances in 2022 and 2023 compared with previous years is not well explained; however, the absence of pest control measures during the COVID-19 pandemic may have played a role in the escalation of cases [18]. In the 1^st^ year of the COVID-19 pandemic (2020), only one case of CCHF was reported, and the identification and reporting of patients with CCHF may have been unintentionally impacted by pandemic-related disruptions in disease monitoring systems and changes in the distribution of healthcare resources. Additionally, changes in agricultural practices and human-animal interactions, possibly caused by socioeconomic changes associated with the pandemic, may have increased exposure to CCHF vectors [19]. The pandemic restrictions and resource shortage in 2020 and the spring of 2021 prevented veterinary departments from conducting scheduled spraying and dipping campaigns, which in turn led to an increase in the tick population [20].

Furthermore, importing animals from nearby countries for Muslim Eids that occurred and/or expanding the number of disease diagnostic tests could have contributed to the rise in CCHF cases reported in the last 2 years. In 2023, the peak number of cases occurred in June and July, which may have been due to more animals (mainly cattle, sheep, and goats) being slaughtered and consumed. Two religious occasions coincided with these 2 months, the first is Eid‐ul‐Adha where many Muslims practice the ritual of Eid al-Adha by slaughtering animals and offering them as a sacrifice and as a gift for the poor and needy persons [21,22]. The second occasion is the Moharram ritual: Millions of Shia Muslims visit the holy places in Karbala and Al Najaf (two provinces in the south of Iraq) during the first 10 days of the Moharram, in 2023, more than five million people visited Karbala during Moharram [23]. Before religious occasions began, many animal owners from rural areas moved to urban areas to sell their animals. Diseases spread to urban areas as a result of these migratory activities, which remain uncontrolled and do not include health inspections of the animals being moved. A few other factors that contribute to the zoonotic transmission of CCHF to the public are independent contractors and nonprofessional slaughterers, lack of butchery training and awareness, lax regulations regarding the sale of animals, animal slaughter in public areas, gatherings near slaughtered animals, improper disposal techniques, and handling of animal blood, tissues, and skin [24,25]. Moreover, In general, Muslims slaughter animals in their homes by themselves, a relative, or a friend, synchronously, the number of slaughtering activities increased by more than 45% during the Eid‐ul‐Adha [26], In addition to the heavy spread of ticks occurred because of the absence of livestock spraying campaigns during the COVID 19 pandemic in 2020 and 2021 [15].

In Muslim-populated areas, epidemiological studies, and historical data have connected Eid-al-Adha celebrations to CCHF, after the celebration, several nearby nations, including Turkey and Iran, have reported serious outbreaks [27]. In agreement with this, Butt et al. found that the outbreak of CCHF during Eid‐Ul‐Adha is considered the most susceptible time for disease contraction in Pakistan, on this occasion, almost five million animals are sacrificed across the country [21,27,28]. Dogan et al. found that most cases of CCHF in Turkey from 2020 to 2022 occurred from June to July, in addition to the fact that the festival will occur in the summer season when animals are more prone to be viremic and infectious of CCHFV, as ticks are more likely to be or have recently been feeding on these animals [29]. On the flip side, one possible explanation for the high number of cases in southern Iraq, particularly in provinces that border Iran, is the illegal cross-border trade in animals. Controlling the spread of zoonotic diseases, such as CCHF, requires reining in illegal trading activities [19].

This study showed that in 2023, up to 58% of cases were male, and up to half of them were residents in rural areas. This could be because they were more involved in farming and shepherding, so they are at high risk for tick bites, same results found by Al Salihi et al., Alhilfi et al., Syed et al., Dogan et al., and Sahak et al. [9,18,[28], [29], [30]]. Our results also showed that approximately one-third (37%) were housewives, 21% were butchers or animal owners, and 23% had other occupations, whereas Alhilfi et al. found that only 13.2% of cases were housewives and 10.8% of butchers or animal owners 57.5% had other occupations [18]. In a study conducted by Sahak et al. in Afghanistan from 2016 to 2018, they found that most of those reported were butchers, animal owners (19%), and housewives (15%), and occupation was not recorded in 26% of cases [30]. Housewives might be at greater risk and more vulnerable because they are in contact with the blood of animals while preparing meals and do not use protective methods. In this study, the confirmed cases had a history of exposure to one or two risk factors up to 60% (328) of cases had a history of exposure to fresh raw meat this might be explained that a high proportion of confirmed cases were housewives, only 8% (43) had history of tick bite, the explanation of low rate of tick bite history may due to bites of these ticks (Hyalomma spp) are usually painless because many people don't even notice their bites. However, we cannot rule out the possibility of tick bites, as they can be painless and go undetected [31], In contrast, other studies have reported results similar to our study or even a lower rate of tick bite history. Sahak et al. conducted a study on 839 detected cases of CCHF in Afghanistan during the period 2016-2018, they found that 12% of reported cases were bitten by a tick, 4% did not know, and the remaining 83% were not bitten by a tick [30]. In a study conducted by Al-Abri et al. in Oman, among 88 cases of CCHF between 1995 and 2017, the major risk factor for infection was contact with animals and/or butchering in 73/88 (83%) cases, and only one case was related to tick bites alone [32]. In contrast, two studies conducted in Turkey by Dogan et al. and Leblebicioglu et al. found that 63% and 69 %, respectively, had a history of tick bites [29,33]. More than half of the cases had a history of slaughtering or were engaged in the slaughtering process, which means that many people in Iraq may slaughter animals such as sheep, goats, and cattle at home outside abattoirs and even without official permission from the veterinary directorate to practice this profession, which directly increases the risk of zoonotic diseases such as CCHF. Furthermore, the General Veterinary Company of the Ministry of Agriculture reported that approximately 7000 unorganized slaughterhouses are distributed throughout the provinces of Iraq [34].

The main limitation of this study was the lack of detailed medical histories and information on all confirmed cases, which restricts the description of all cases. These constraints have been present in the past. In a study by Mustafa et al. of 143 suspected cases in 2018, they found that only 31.5% of suspected cases that were finally identified by the CDC's Iraqi zoonotic disease section met the standard case definition, and 68.5% of suspected cases did not, instead relying solely on one symptom—either fever or the presence of hemorrhagic symptoms–with or without a history of animal contact. This information was based on the case investigation forms for all suspected cases. Furthermore, a number of the checklist forms' areas, such as risk factors, clinical indications and symptoms, educational background, and socioeconomic level, were not accurately filled out [35]. In 2023, up to 92% of the cases (539 of 587) will have complete data, and more actions are needed to improve the reporting process of suspected and confirmed cases.

## Conclusion

The CCHF outbreak in 2023 was the largest in Iraq in decades. The absence of preventive and control activities during the COVID-19 pandemic played an important role in the rise of cases in 2022-2023 and the presence of an unofficial slaughtering process of animals by unlicensed and freelance slaughterers, especially during religious events played an important role in this epidemic. To control the zoonotic transmission of CCHF to the general public, many factors that play an important role in the transmission mechanism should be controlled, including the use of unlicensed and freelance slaughterers; lack of butchery training and awareness; inadequate regulations on animal sales; animal slaughter in public areas; gatherings around slaughtered animals; improper disposal procedures; and the handling of animal blood, tissues, and skin. Animal health checks should be regulated to reduce tick infections in cattle. The waste and blood of sacrificed animals should be disposed of using appropriate methods such as rendering, landfilling, composting, and anaerobic digestion. Therefore, animal movement across borders should be controlled. Social mobilization and community engagement are key to controlling the transmission of CCHF; however, no remarkable campaigns have been initiated by officials to enhance public awareness of CCHF.

## Declarations of competing interest

The authors have no competing interests to declare.

## Appendix Supplementary materials

Image, application 1
