# Supplementary material for: Crimean-Congo hemorrhagic fever, a real health problem in Iraq?
Source: IJID Reg. 2025 Jan 31;14:100588. doi: 10.1016/j.ijregi.2025.100588 (PMC11874820; doi:10.1016/j.ijregi.2025.100588)
Supplement: Supplementary file 1 [file mmc1.docx]

Supplementary materials


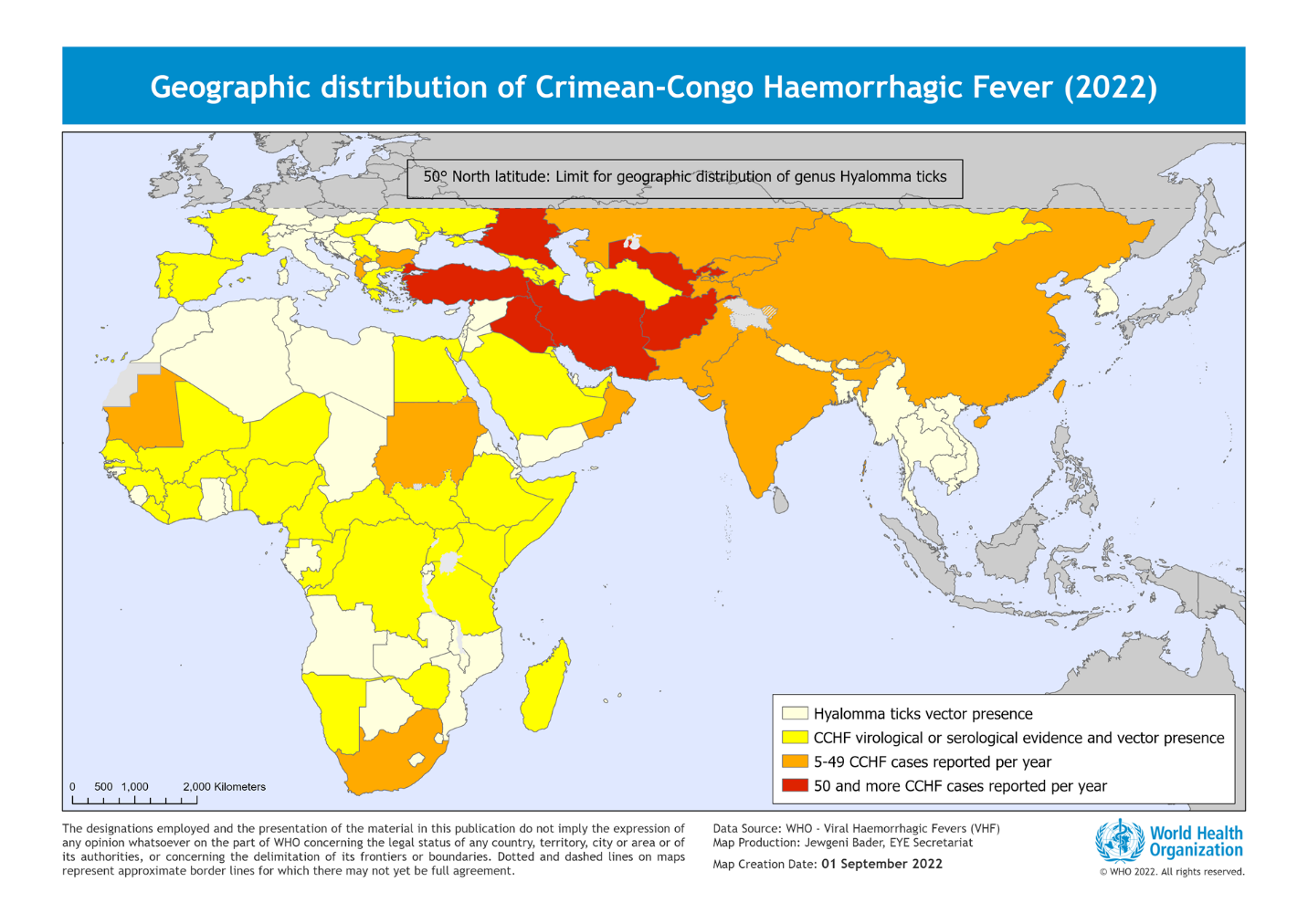


Figure 1: Geographic distribution of CCHF and Hyalomma spp. Ticks[2]


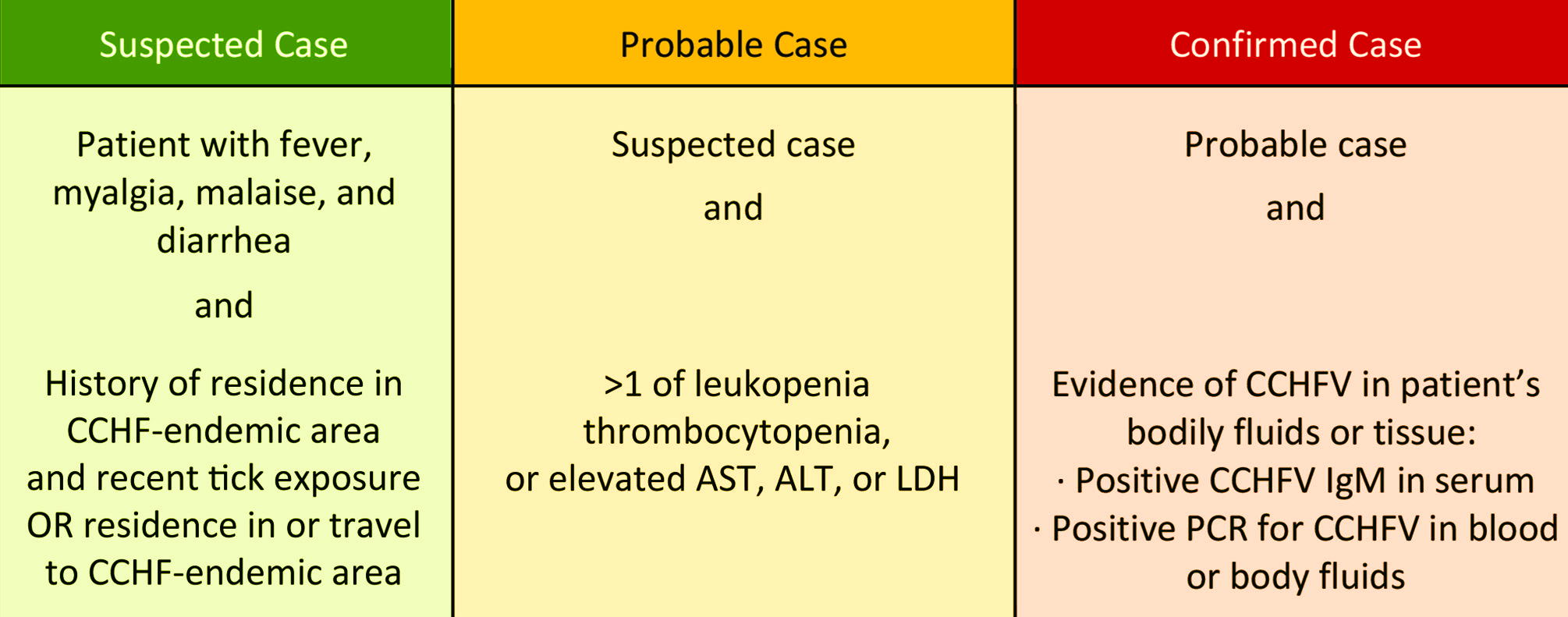


Figure 2: CCHF case definitions, modified from Ergonul et al.[11-12]
